# Data from timing fear cues in the temporal bisection task

**DOI:** 10.1016/j.dib.2019.104491

**Published:** 2019-09-06

**Authors:** Erich K. Grommet, Nancy S. Hemmes, Bruce L. Brown

**Affiliations:** Queens College and the Graduate Center, City University of New York, USA

**Keywords:** Temporal bisection, Timing, Emotion

## Abstract

Research on emotion often involves the use of emotion-evoking stimuli that are used to manipulate emotional state across groups or conditions. One standardized set of stimuli that has been used for this purpose is the International Affective Picture System (IAPS) [1]. The data described in this article were obtained over the course of two experiments in which the primary task was for participants to judge the presentation duration of six IAPS pictures in the temporal bisection task [2–4]. Each of these experiments contained three types of phases (rating, training, and testing). In rating phases, participants rated the IAPS pictures for evoked valence, arousal, and fear. In training phases, participants were trained to classify the presentation duration of green squares (Experiment 1) or IAPS pictures (Experiment 2) as either “short” or “long.” In testing phases, participants were instructed to use what they had learned in the preceding training phases to classify the IAPS pictures as either “short” or “long.” The findings related to these data were published in Grommet, Hemmes, and Brown [5], and the data are available in Mendeley Data, DOI: 10.17632/xx6zh6mmjw.1 [6].

**Table:** Specifications Table

Subject area	Psychology
More specific subject area	Temporal Perception
Type of data	Comma delimited text file
How data were acquired	Participants entered responses on a microcomputer
Data format	Raw
Experimental factors	Temporal bisection task data were collected from introductory psychology students using images from the International Affective Picture System (IAPS) [1].
Experimental features	Rating, training, and testing data
Data source location	Flushing, NY, Queens College, City University of New York
Data accessibility	Data are available in Mendeley Data, https://doi.org/10.17632/xx6zh6mmjw.1[6]
Related research article	Grommet, E. K., Hemmes, N. S., and Brown, B. L. (in press). The role of clock and memory processes in the timing of fear cues by humans in the temporal bisection task. *Behavioural Processes.*[5]

**Table:** 

**Value of the data** • Access to the testing phase data allows researchers to calculate the temporal bisection point using the method employed in their laboratory, thus allowing for better comparison of data generated from different laboratories. • Data on latency to respond, which may have implications for understanding participant decision making in the temporal bisection task, are included in the datasets. These data are generally not published and, therefore, not widely available for inspection. • Access to these data allows for the calculation and, therefore, inspection of the effect of manipulations at the individual-subject level.

## 1 Data

Rating, training, and testing phase data were collected in a temporal bisection task [2], [3], [4]. The first four columns in each dataset are subject number, sex, sequence group, and session number. These common identifiers allow researchers to link a given subjects data across phases or sessions of an experiment for within-subjects analyses or isolate analyses to a given sex, sequence group, or session while including data from more than one phase of the experiment. The remaining columns in the rating phase datasets (Experiment 1: rating-3DR.csv, Experiment 2: rating-MM.csv) are test (pretest vs. posttest), rated stimulus, and participant ratings of the stimuli (valence, arousal, and fear). The remaining columns in the training phase datasets (Experiment 1: training-3DR.csv, Experiment 2: training-MM.csv) are timed stimulus, timed stimulus duration, participant response (“short” vs. “long”), response feedback (“correct” vs. “incorrect”), and response latency from stimulus offset. The remaining columns in the rating phase datasets (Experiment 1: testing-3DR.crv, Experiment 2: testing-MM.crv) are trial block (fist vs. second 84 trials in session), timed stimulus, timed stimulus duration, fixation point duration, participant response (“short” vs. “long”), and response latency from stimulus offset. See Table 1 for a detailed description of the columns in each datafile type.

**Table 1:** Explanation of each column in the data files by phase.

Phase	Column name	Description
rating	participant_number	Participant's unique identification number.
	sex	Participant's sex. M = male, F = female.
	sequence_group	Experiment 1: Order participants were exposed to duration ranges. SML, SLM, MSL, MLS, LSM, LMS. S = 250–1000 ms, M = 400–1600 ms, L = 550–2200 ms. Experiment 2: Order participants were exposed to session types. FNM, FMN, NFM, NMF, MFN, MNF. F = fear only, N = neutral only, M = mixed.
	session_number	1 = first session, 2 = second session, 3 = third session.
	test	A = pretest, B = posttest.
	rated_stimulus	IAPS pictures. 1052 = snake, 1321 = bear, 1931 = shark, 7010 = basket, 7020 = fan, 7175 = fan.
	valence_rating	1-9 scale. 1 = negative, 9 = positive.
	arousal_rating	1-9 scale. 1 = calm, 9 = excited.
	fear_rating	1-7 scale. 1 = no fear, 7 = fear.
training	participant_number	Participant's unique identification number.
	sex	Participant's sex. M = male, F = female.
	sequence_group	Experiment 1: Order participants were exposed to duration ranges. SML, SLM, MSL, MLS, LSM, LMS. S = 250–1000 ms, M = 400–1600 ms, L = 550–2200 ms. Experiment 2: Order participants were exposed to session types. FNM, FMN, NFM, NMF, MFN, MNF. F = fear only, N = neutral only, M = mixed.
	session_number	1 = first session, 2 = second session, 3 = third session.
	timed_stimulus	Experiment 1: green = green square. Experiment 2: IAPS pictures. 1052 = snake, 1321 = bear, 1931 = shark, 7010 = basket, 7020 = fan, 7175 = fan.
	timed_stimulus_duration	Presented in ms.
	participant_response	S = short; L = long.
	response_feedback	correct, incorrect.
	response_latency	Presented in ms.
	
testing	participant_number	Participant's unique identification number.
	sex	Participant's sex. M = male, F = female.
	sequence_group	Experiment 1: Order participants were exposed to duration ranges. SML, SLM, MSL, MLS, LSM, LMS. S = 250–1000 ms, M = 400–1600 ms, L = 550–2200 ms. Experiment 2: Order participants were exposed to session types. FNM, FMN, NFM, NMF, MFN, MNF. F = fear unmixed, N = neutral unmixed, M = mixed.
	session_number	1 = first session, 2 = second session, 3 = third session.
	trial_block	1 = first 84 trials, 2 = second 84 trials.
	timed_stimulus	IAPS pictures. 1052 = snake, 1321 = bear, 1931 = shark, 7010 = basket, 7020 = fan, 7175 = fan.
	timed_stimulus_duration	Presented in ms.
	fixation_point_duration	Presented in ms.
	participant_response	S = short; L = long.
	response_latency	Presented in ms.

## 2 Experimental design, materials, and methods

### 2.1 Experiment 1

*Participants.* Forty-eight Queens College, City University of New York (CUNY), introductory psychology students (35 female and 13 male) served as participants. The protocol was approved by CUNY's Human Research Protection Program (HRPP).

*Materials.* Stimuli consisted of six pictures from the International Affective Picture System (IAPS) [1]. Three pictures had the highest normative arousal ratings out of the pictures that evoked the single emotion category of fear, as per the criteria used in Mikels et al. [6] (snake, bear, and shark; IAPS numbers 1052, 1321, and 1931 respectively). The three other pictures had normative ratings that indicated neutrality, i.e., low arousal ratings and valence ratings near the midpoint between positive and negative (basket-7010, fan-7020, and lamp-7175).

*General procedure.* Each participant was exposed to three sessions that each corresponded to one of the following duration ranges: 250-100 ms, 400–1600 ms, or 550–2200 ms. A session began and ended with a rating phase (i.e., pretest and posttest). Between the two rating phases, there was a training phase followed by a testing phase.

*Rating phase.* Participants were exposed to all six IAPS pictures (three fear-evoking and three neutral). Prior to each picture presentation, a fixation point was presented for 800 ms. Each picture was shown for 1225 ms. Following the presentation of each picture, participants rated the picture in regard to evoked valence, arousal, and fear.

*Training phase.* Participants were exposed to presumably innocuous green squares that were presented in the center of the screen for either the short or long anchor duration of the range that the participants were exposed to in the ensuing testing phase of the session. Prior to each square presentation, a fixation point was presented for 800 ms. The task of the participants was to press the “S” or the “L” key on the computer keyboard following the presentation of each square to indicate whether the square was presented for the short or long duration, respectively. Following each response, feedback (i.e., “correct” or “not correct”) was presented for 2000 ms. Blocks of eight green squares (four short and four long, in random order) continued to be presented until the participant completed a block with no errors.

*Testing phase.* Participants were exposed to two blocks of 84 trials, for a total of 168 trials per session. Each block contained 12 picture presentations at of each of the seven durations that were comprised of the two anchor durations used in the training phase and five linearly-spaced intermediate probe durations. Each of the six IAPS pictures was presented twice at each duration in each block, and the presentation sequence of these picture-duration combinations was random within each block. Prior to each picture presentation, a fixation point was presented for 1300–3300 ms (randomly determined). The task of the participants was to press the “S” or the “L” key on the computer keyboard following each picture presentation to indicate whether the picture was presented for a short or long duration, respectively. No feedback was given in this phase, and it ended following the response to the last picture presentation.

### 2.2 Experiment 2

*Participants***.** Forty-eight Queens College, CUNY, introductory psychology students (36 female and 12 male) served as participants. None of these students participated in Experiment 1. This experiment was approved by CUNY's HRPP.

*Materials.* Experiment 2 used the same materials as Experiment 1. The only exception was that IAPS pictures replaced the green square in the training phase.

*General Procedure.* Each participant was exposed to three sessions that each corresponded to one of the following session types: fear-evoking IAPS pictures, neutral IAPS pictures, or a mixture of fear-evoking and neutral IAPS pictures. A session began and ended with a rating phase (i.e., pretest and posttest). Between the two rating phases, there was a training phase followed by a testing phase.

*Rating phase.* The procedure for the rating phase was the same as in Experiment 1, except that each picture was shown for 1375 ms.

*Training phase.* IAPS pictures were presented for either the shortest (550 ms) or longest (2200 ms) duration to which the participants were exposed in the testing phase. In the fear-unmixed session, each of the three fear-evoking pictures was shown two times at each of the two anchor durations per training block. In the neutral-unmixed session, each of the three neutral pictures was shown two times at each of the two anchor durations per block, and in the mixed session, all six pictures were shown one time at each duration per block. The fixation point procedure, participant task, and feedback procedure were the same as in the training phase of Experiment 1. Blocks of twelve pictures (six short and six long, in random order) continued to be presented until the participant completed a block with no errors.

*Testing phase.* Participants were exposed to two blocks of 84 trials (168 trials in total) per session. Each block contained 12 presentations of each of the two durations used in the training phase (anchor durations) and of the five linearly-spaced intermediate probe durations that fell between the two training durations. Each of the three fear pictures was presented four times per block at each of the seven durations in the fear-unmixed session, each of the three neutral pictures was presented four times per block at each of the seven durations in the neutral-unmixed session, and each of the six pictures was presented two times per block at each of the seven durations in the mixed session. The fixation point procedure and participant task were the same as in the testing phase of Experiment 1.
